# Supplementary material for: Structure of the human systemic RNAi defective transmembrane protein 1 (hSIDT1) reveals the conformational flexibility of its lipid binding domain
Source: Life Sci Alliance. 2024 Jun 26;7(9):e202402624. doi: 10.26508/lsa.202402624 (PMC11208740; doi:10.26508/lsa.202402624)
Supplement: Supplementary file 1 [file LSA-2024-02624_TableS1.docx]

**Table S1: Comparison of the sample preparation conditions employed by various studies**

|  | **Qian et al.,** [33] | **Zheng et al.,** [36] | **Hirano et al.,** [35] | **Sun et al.,** [34] | **Liu et al., [37]** | **This study** |
| --- | --- | --- | --- | --- | --- | --- |
|  |  | **Expression** | | | | |
| **Cell type** | HEK293F | Sf9 | Expi293F | HEK293F | HEK293F | HEK293sGnTI- |
| **Expression construct** | Full-length hSIDT2 | ΔCL1-hSIDT1 and ΔCL1-hSIDT2 | Full-length hSIDT1 | Full-length hSIDT1 | Full-length hSIDT1 | Full-length hSIDT1-GFP |
| **Gene transfer** | Transient transfection | Baculoviral transduction | Baculoviral transduction | Transient transfection | Baculoviral transduction | Baculoviral transduction |
| **Cell culture time/temp** | 48 h/37 ^o^C | 60 h/27 ^o^C | 96 h/30^o^C | 12 h/37 ^o^C  48 h/30 ^o^C | 12 h/37 ^o^C  36 h/30 ^o^C | 8 h/37 ^o^C  36 h/32 ^o^C |
| **Culture volume** | 12 L | 6 L | - | 1L | - | 1 L |
|  |  | **Purification** | | | | |
| **Cell lysis buffer** | 25 mM HEPES,  pH 7.4, 150 mM NaCl, 1.95 μg/ml aprotinin, 1.5 μg/ml  pepstatin, and 3 μg/ml leupeptin | 50 mM HEPES, pH 7.5, 300 mM NaCl, 1.04 mM AEBSF, 0.8 μM Aprotinin, 50 μM Bestatin, 15 μM E-64, 20 μM Leupeptin, 15 μM Pepstatin A, and 1 mM PMSF | 20 mM Tris-HCl, pH 7.0, 150 mM NaCl, and 1X protease inhibitor cocktail | 40 mM Tris-HCl, pH 7.5, 150 mM NaCl, 20% glycerol (v/v), and 1X protease inhibitor cocktail | 25 mM HEPES, pH 7.5, 250 mM NaCl, 5% glycerol | 25 mM Tris, pH 7.5, 200 mM NaCl, 0.8 μM aprotinin, 2 μg/ml leupeptin, and 2 μM pepstatin A |
| **Cell lysis** | - | High-pressure cell disruption | Sonication | High-pressure Homogenizer | Sonication | Sonication |
| **Detergent for solubilization prepared in cell lysis buffer** | 1% (w/v) DMNG and 0.1% (w/v) CHS | 2% (w/v) DDM and 0.2% (w/v) CHS | 1.7% (w/v) LMNG or digitonin | 1% (w/v) LMNG and 0.1% (w/v) CHS | 1 % (w/v) DDM and 0.2% (w/v) CHS | 1% (w/v) Digitonin |
| **Affinity resin** | Tandem anti-FLAG & Ni-NTA | Anti-FLAG | Anti-FLAG | Anti-FLAG | Strep-Tactin | Strep-Tactin |
| **Wash buffer** | 0.01% GDN in cell lysis buffer | 0.02% (w/v) LMNG  0.002% (w/v) CHS in cell lysis buffer, plus 1 mM EDTA | 0.01% LMNG or 0.01% GDN 20 mM Tris-HCl, pH 7.0, and 150 mM NaCl | 40 mM Tris-HCl, pH 7.5, 150 mM NaCl, 10% glycerol (v/v), 1 mM DTT, 0.5 mM ATP-Mg^+2^, 0.02% (w/v) GDN | 0.01% GDN, 25 mM HEPES, pH 7.5, 250 mM NaCl, 5% glycerol | 0.5% (w/v) Digitonin, 25 mM Tris, pH 7.5, and 200 mM NaCl |
| **Elution buffer** | 0.01% GDN in cell lysis buffer, plus 300 ug/ml FLAG peptide (for Anti-FLAG) or 300 mM imidazole for Ni-NTA) | 0.01% (w/v) LMNG  0.001% (w/v) CHS in cell lysis buffer, plus 500 ug/ml FLAG peptide, 50 mM HEPES, pH 7.5, and 300 mM NaCl | 0.01% LMNG or 0.01% GDN 20 mM MES-NaOH, pH 6.0, 150 mM NaCl, and 5 M LiCl | 40 mM Tris-HCl, pH 7.5, 150 mM NaCl, 10% glycerol (v/v), 1 mM DTT, 0.02% (w/v) GDN, plus 200 ug/ml FLAG peptide | 5 mM Desthiobiotin in wash buffer | 5 mM Desthiobiotin in wash buffer |
| **SEC buffer** | 0.006% (w/v) GDN, 25 mM HEPES, pH 7.4, and 150 mM NaCl | 0.01% (w/v) LMNG  0.001% (w/v) CHS,  25 mM HEPES, pH 7.5, 150 mM NaCl, and 0.5 mM EDTA | 0.01% LMNG or 0.01% GDN 20 mM MES-NaOH, pH 6.0, and 150 mM NaCl | 40 mM Tris-HCl, pH 7.5, 150 mM NaCl, and 0.02% GDN | 0.005% GDN, 25 mM HEPES, pH 7.5, 100 mM NaCl. For pH 5.5, 25 mM MES was used instead of HEPES | 0.5% (w/v) Digitonin, 25 mM Tris, pH 7.5, and 200 mM NaCl |
| **Protein concentration** | 13 mg/ml | 3.5 mg/ml | 2.5-5.0 mg/ml | 17 mg/ml | 10 mg/ml | 1 mg/ml |
|  |  | **Cryo-EM sample** | | | | |
| **Grid type** | Quantifoil Au 300 mesh, R1.2/1.3 | Quantifoil Cu 300 mesh, R0.6/1.0 or Nanodim R1.2/1.3 amorphous nickel titanium alloy (ANTA) grid | Quantifoil Cu 300 mesh, R1.2/1.3 | Quantifoil Cu 300 mesh, R1.2/1.3 | Ni-Ti Au 300 mesh (Nanodim Tech). | UltrAuFoil Au 300 mesh, R1.2/1.3 |
| **Pretreatments** | Grids glow-discharged | Grids glow-discharged and treated with 0.1% poly L-lysine hydrobromide | Grids glow-discharged | Grids glow-discharged | Grids glow-discharged | Grids glow-discharged, and 100 μM FOM added to protein sample |
| **Blotting** | 3 s, 8 ^o^C,  100% humidity | 2.5-3 s, 8 ^o^C, 100% humidity | 4 s, 6 ^o^C,  100% humidity | 4s, 6 ^o^C,  100% humidity | 3s, 4 ^o^C,  100% humidity | 2.5 s, 18 ^o^C, 100% humidity |
|  |  | **Cryo-EM data collection** | | | | |
| **Microscope** | Titan Krios (K3, 300 kv) | Titan Krios (K2/K3, 300 kv) | Titan Krios (K3, 300 kv), CDS mode | Titan Krios (K3, 300 kv) | Titan Krios (K3, 300 kv) | Titan Krios (K3, 300 kv) |
| **Pixel size** | 1.08 Å/px | 0.82 Å/px | 0.83 Å/px | 0.535 Å/px | 0.85 Å/px | 0.85 Å/px |
| **Total dose** | 50 e^-^/Å^2^ | 60 e^-^/Å^2^ | 66 e^-^/Å^2^ | 50-60 e^-^/Å^2^ | 52 e^-^/Å^2^ | 50 e^-^/Å^2^ |
| **Defocus range** | -1.3 to -1.8 um | -1.2 to -1.8 um | - | -1.5 to -2.0 um | -1.0 to -1.5 um | -1.0 to -2.5 um |
