# Supplementary figures and images for: Structure of the human systemic RNAi defective transmembrane protein 1 (hSIDT1) reveals the conformational flexibility of its lipid binding domain
Source: Life Sci Alliance. 2024 Jun 26;7(9):e202402624. doi: 10.26508/lsa.202402624 (PMC11208740; doi:10.26508/lsa.202402624)

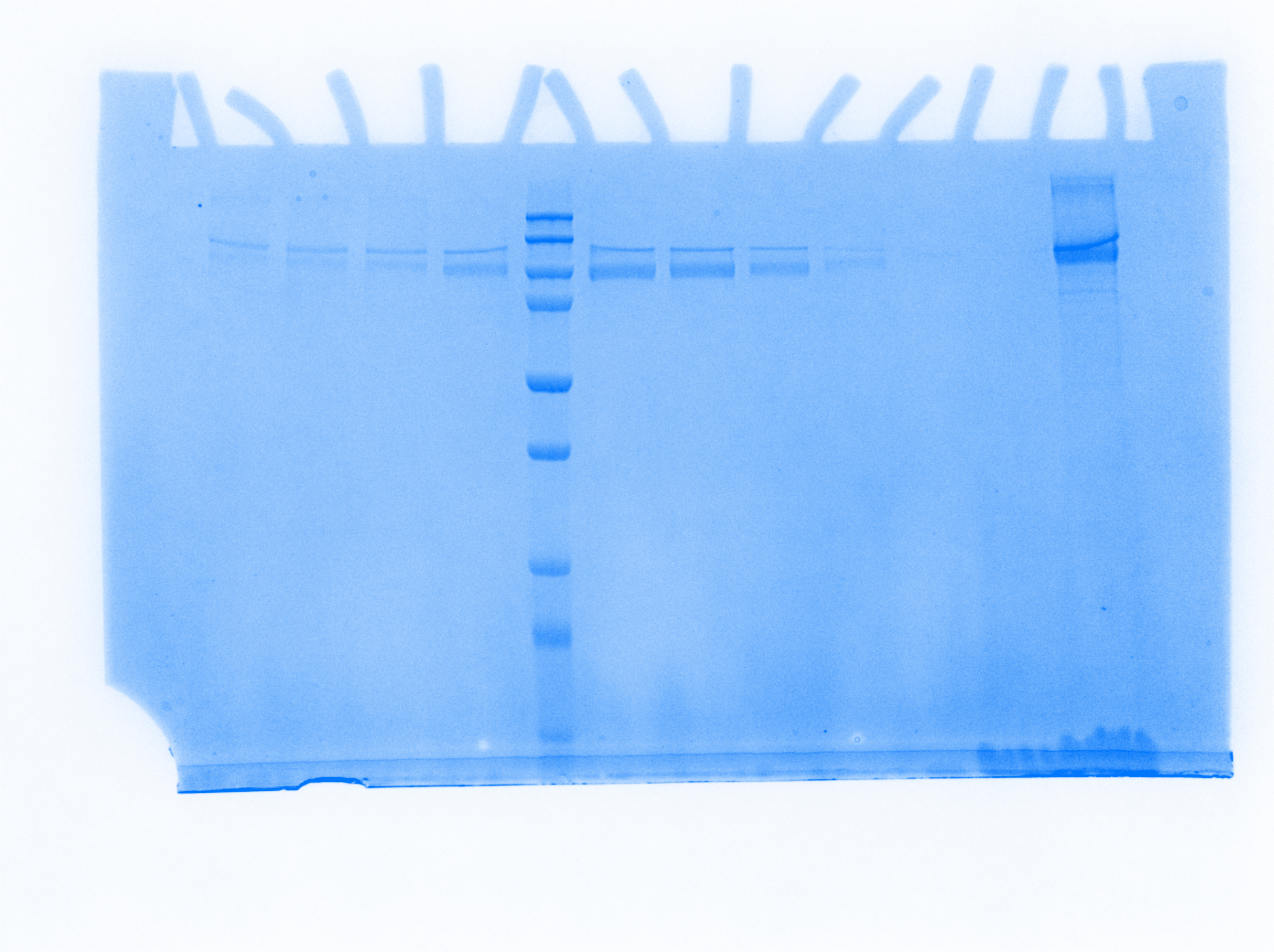

Supplement: Supplementary file 2 [file LSA-2024-02624_SdataFS1.1.tif]
