# Supplementary material for: Structure of the human systemic RNAi defective transmembrane protein 1 (hSIDT1) reveals the conformational flexibility of its lipid binding domain
Source: Life Sci Alliance. 2024 Jun 26;7(9):e202402624. doi: 10.26508/lsa.202402624 (PMC11208740; doi:10.26508/lsa.202402624)
Supplement: Supplementary file 4 [file LSA-2024-02624_TableS2.docx]

**Table S2: Comparison of the Cα RMSDs of hSIDT1-GFP (8V38) superposition on the existing ChUP family structures**

|  | **8JUL** | **8JUN** | **8HIIP** | **8HKE** | **7Y63** | **7Y68** | **7Y69** | **8WOT** | **8WOR** |
| --- | --- | --- | --- | --- | --- | --- | --- | --- | --- |
| **ECD dimer Cα RMSD** | 1.2 Å | 1.2 Å | 6.7 Å | 7.0 Å | 2.2 Å | 1.5 Å | 1.4 Å | 0.9 Å | 0.9 Å |
| **TMD dimer Cα RMSD** | 4.2 Å | 2.9 Å | 6.0 Å | 5.7 Å | 3.6 Å | 3.4 Å | 3.5 Å | 2.9 Å | 3.8 Å |
| **Full dimer Cα RMSD** | 4.1 Å | 3.1 Å | 8.2 Å | 9.0 Å | 3.3 Å | 3.3 Å | 3.3 Å | 2.6 Å | 3.3 Å |
| **ECD chain A Cα RMSD** | 1.2 Å | 1.2 Å | 6.7 Å | 9.9 Å | 2.2 Å | 1.5 Å | 1.5 Å | 0.9 Å | 0.9 Å |
| **TMD chain A Cα RMSD** | 4.2 Å | 2.9 Å | 6.0 Å | 5.7 Å | 3.6 Å | 3.4 Å | 3.4 Å | 2.8 Å | 3.9 Å |
| **Full chain A Cα RMSD** | 4.1 Å | 3.2 Å | 8.2 Å | 9.0 Å | 3.3 Å | 3.3 Å | 3.3 Å | 2.6 Å | 3.4 Å |
